# Flares in Lupus Nephritis: Risk Factors and Strategies for Their Prevention

**DOI:** 10.1007/s11926-023-01109-6

**Published:** 2023-07-15

**Authors:** Aggelos Banos, George Bertsias

**Affiliations:** 1 Department of Rheumatology, ‘Asklepieion’ General Hospital, Voula, Athens, Greece; 2 Laboratory of Autoimmunity and Inflammation, Center of Clinical, Experimental Surgery and Translational Research, Biomedical Research Foundation Academy of Athens, 115 27, Athens, Greece; 3 Rheumatology, Clinical Immunology and Allergy, University Hospital of Heraklion and University of Crete Medical School, Voutes-Stavrakia, 71008 Heraklion, Greece; 4 Institute of Molecular Biology and Biotechnology-FORTH, Heraklion, Greece

**Keywords:** Systemic lupus erythematosus, End-stage kidney disease, Risk stratification, Therapeutic target, Flares, Biologic agents

## Abstract

**Purpose of Review:**

Discuss the prognostic significance of kidney flares in patients with lupus nephritis, associated risk factors, and possible preventative strategies.

**Recent Findings:**

Recently performed clinical trials and observational cohort studies underscore the high frequency of relapses of kidney disease, following initial response, in patients with proliferative and/or membranous lupus nephritis. Analysis of hard disease outcomes such as progression to chronic kidney disease or end-stage kidney disease, coupled with histological findings from repeat kidney biopsy studies, have drawn attention to the importance of renal function preservation that should be pursued as early as lupus nephritis is diagnosed. In this respect, non-randomized and randomized evidence have suggested a number of factors associated with reduced risk of renal flares such as attaining a very low level of proteinuria (< 700–800 mg/24 h by 12 months), using mycophenolate over azathioprine, adding belimumab to standard therapy, maintaining immunosuppressive/biological treatment for at least 3 to 5 years, and using hydroxychloroquine. Other factors that warrant further clarification include serological activity and the use of repeat kidney biopsy to guide the intensity and duration of treatment in selected cases.

**Summary:**

The results from ongoing innovative studies integrating kidney histological and clinical outcomes, together with an expanding spectrum of therapies in lupus nephritis, are expected to facilitate individual medical care and long-term disease and patient prognosis.

## Introduction

Undeniably, kidney disease represents a hallmark of systemic lupus erythematosus (SLE), imposing significant consequences on patients at a personal, medical, and societal level. Contemporary worldwide trends of biopsy-proven lupus nephritis (LN) approximate 30% [1] with adjusted incidence rates of 0.60 per 100,00 adults/year [2]. Although often appearing as a presenting SLE manifestation, an increasing number of LN cases develop during the disease course [3], which might be related to the earlier diagnosis and increased recognition of milder forms of lupus. In the same context, an observational study covering the period 1970–2016 indicated that despite the decreasing occurrence of rapidly progressive glomerulonephritis, proliferative (class III or IV) forms are still the predominant histological type, whereas pure membranous (class V) form accounts for 16–20% of incident LN cases [4].

Despite significant advances in LN including the publication of high-quality management recommendations, the definition of treatment goals associated with improved outcomes, and the approval of novel therapies [5–8], SLE patients with kidney involvement are still burdened with increased morbidity and mortality [9]. Moreover, it is worrisome that rates of lupus-associated end-stage kidney disease (ESKD) are stable, non-improving during the last 2 decades [10, 11]. Although several factors may contribute to these trends, exacerbations (flares) of renal activity are a well-recognized driver for adverse kidney and patient prognosis. Herein, we review the frequency and clinical significance of LN flares, focusing on pertinent intrinsic and extrinsic risk factors. Importantly, we discuss strategies that can potentially facilitate durable renal response thus, improving long-term outcomes.

## Flares Are Common Events in the Course of Lupus Nephritis

SLE is an archetypal waxing-waning autoimmune disorder, so flares are inherent to the natural history of the disease [12, 13]. In this respect, following the initial response, re-activation of kidney disease may occur at a frequency that may depend on a variety of factors. Although there is heterogeneity in the definitions of renal flares, these are typically based on a combination of increases in urine protein excretion, hematuria or urine sediment, and/or lowering of renal function [13]. The traditional distinction includes proteinuric flares characterized by increases in proteinuria (usually to a level exceeding 2 g/24 h) with no significant change in estimated glomerular filtration rate (eGFR; stable at less than 30% increase over baseline), and absent or minimal glomerular hematuria. On the other hand, nephritic flares are accompanied by increases in proteinuria, glomerular hematuria (by at least twofold or above 10 red blood cells/hpf), re-appearance of urine casts, with or without deterioration in eGFR (at least a 30% increase in serum creatinine) in cases of severe flares [13].

Although not evaluated as the primary endpoint in LN clinical trials, analysis of flares has been used to complement response rates and determine the relative efficacy of new therapies over standard-of-care. In the MAINTAIN study, LN patients received low-dose intravenous cyclophosphamide and then continued with either mycophenolate or azathioprine [14]. Relapses occurred at 30–40% within the first 2–4 years. In the BLISS-LN trial, patients randomized on the standard treatment arm (either low-dose intravenous cyclophosphamide followed by azathioprine, or mycophenolate as both initial and subsequent therapy) experienced relapses at 26.0% rate over a 2-year period [15•]. Notably, the subgroup of patients with class V LN had the highest frequency of flares (34.4%). The exactly same relapse rate (26.0%) was reported for the control group (mycophenolate) in the long-term extension phase of the AURORA 1 and AURORA 2 trials [16].

Real-world evidence from recent patient cohorts also uncovers a substantial burden of LN flares. Specifically, Pirson et al. [17] analyzed 128 patients with incident LN (class III, IV, or V) followed for a median period of 134 months and reported 32% flares after initial remission. This is comparable to the findings (33.0% flares) from a retrospective analysis of 100 Greek LN patients [18]. In a study by Luis et al. [19], 104 patients with LN were monitored over an average of 34.5 months. The vast majority (91.6%) achieved complete renal response but later, flares developed in 18.4%. In two studies from Eastern Asia, flares were reported at a range of 32.2–37.0% [20, 21]. Finally, Momtaz et al. [22] focused on nephritic flares which occurred in 12.6–27.8% of a very large cohort of LN patients. Collectively, the aforementioned data align with previously reported estimates of kidney relapse-free survival rates of 96%, 90%, 86%, 80%, 69%, and 57% at 1, 2, 3, 4, 5, and 10 years, respectively [23].

## Flares as a Major Determinant of Adverse Outcomes in Lupus Nephritis

In SLE, every flare of disease activity carries an almost twofold increased risk for accrual of irreversible organ damage typically quantified by the SLICC/ACR damage index [24]. In the case of LN specifically, it has long been appreciated that the re-appearance of renal activity is accompanied by progression in kidney histological lesions. Thus, histological class transformation may occur, especially (> 55%) in patients with non-proliferative nephritis in the first biopsy [25]. In addition, repeat biopsies performed in the context of clinically defined flares, usually demonstrate increases in chronicity lesions due to glomerular and/or tubulointerstitial scarring [26•]. These observations underly the concept that each LN flare may reduce the functional reservoir of the kidney, thus precipitating the onset of chronic kidney disease (CKD) and/or ESKD [25, 27]. Indeed, observational studies have illustrated an inverse relationship between the incidence of flares and renal function impairment [28]. In a series of severe LN cases, spending more than 30% of the time under kidney flare had an odds ratio (OR) of 20 (95% confidence interval, 4.6–91.3) for developing new or progressive CKD [29]. Similarly, Perez-Arias et al. [30] demonstrated a reduction of complete and partial renal response rates as well as an excess in kidney and patient survival, both correlating with an increasing number of LN flares.

Notably, nephritic flares are generally considered more deleterious to the kidney with reported hazard ratios ranging 13.6–27.0 for doubling of serum creatinine and/or ESKD [31, 32]. Nonetheless, proteinuric flares, especially when they occur at early time points (≤ 18 months), are also linked to dismal outcomes [33]. Flares of both proliferative and membranous forms of LN may be detrimental to kidney fitness [29].

Additional consequences of LN flares include the negative impact on health-related quality of life [34] and the need for treatment with glucocorticoids, often used at moderate/high dose and/or for a prolonged period of time [5], thus resulting in organ damage accrual. Finally, the economic aspects of LN flares cannot be overemphasized due to significantly increased direct healthcare costs [35–37].

## Individual- and Disease-Inherent Factors Associated with LN Flares

**Fig. 1:**
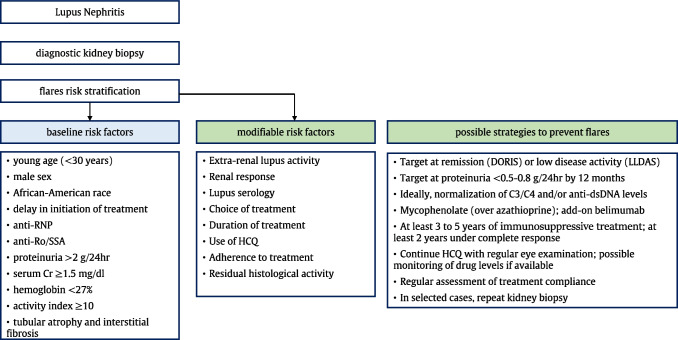
Assessment of the risk for flares in patients with lupus nephritis and possible preventative strategies. Cr, creatinine; HCQ, hydroxychloroquine, DORIS, Definition of Remission in SLE; LLDAS, lupus low disease activity state

In view of the prognostic implications of LN exacerbations, there have been efforts to define subgroups of patients at high risk for flares, as this can facilitate personalized monitoring and the application of preventative and/or early therapeutic approaches. To this end, a variety of demographic, clinical, immunological, histological, and other parameters have been linked to an augmented risk for LN flares. These can be roughly grouped into fixed (i.e., inherent to the patient or the disease) or modifiable factors, the latter creating possibilities for risk-lowering strategies (discussed below) (Fig. 1). Fixed risk factors include the younger age (especially less than 30 years) of the patient [19, 31, 33, 38], male sex [32], African-American race [39], and delay (more than 5 months) in the initiation of immunosuppressive treatment [40]. LN patients with a history of anti-RNP [19] and anti-Ro/SSA [41] autoantibodies, although neither specific to SLE, seem to be at increased risk for relapses.

In terms of clinical characteristics at the time of diagnosis, observational studies have identified increased proteinuria (above 2 g/24 h) [18], serum creatinine ≥ 1.5 mg/dL [31, 32], and anemia (hemoglobin below 27% [32]) as predictors for flares following the initial response to treatment. Finally, certain histological features—all signifying disease aggressiveness—are associated with more frequent relapses. These include a high NIH activity index (≥ 10) [33], the presence of tubulointerstitial inflammation [42], karyorrhexis and endocapillary hypercellularity [33, 38], and finally, chronic lesions such as tubular atrophy and interstitial fibrosis [18, 42]. Most of the aforementioned predictors are associated with a 1.5–2.5-fold risk for flares; however, these associations have not always been adjusted for possible confounding.

## Modifiable Risk Factors and Preventive Strategies for Lupus Nephritis Flares (Fig. 1)

### Extra-Renal Lupus Activity

Although LN patients often present with so-called “organ-dominant” disease, it is not uncommon that other lupus features are present either at the onset or during the course of the disease. Extra-renal disease manifestations may become more apparent or symptomatic when treatment (especially glucocorticoids) is reduced or modified to maintenance dosages or regimens. For example, in our experience, although mycophenolate is generally effective at maintaining a renal response, it may not always suffice to control residual arthritis or skin rashes. Circumstantial evidence suggests that persistent SLE activity can precipitate a flare from a major organ such as the kidney [32] or the central nervous system [43]. Notably, a study in pediatric LN cases showed that achievement of lupus low disease activity state (LLDAS, an established treatment target for general SLE [44]) after initial treatment and LLDAS-50 (i.e., LLDAS attained for at least 50% of the total observation period) were both associated with significantly lower rates of kidney flare [45]. Importantly, due to the advent of novel biological treatments such as belimumab, management of extra-renal disease activity may not necessarily mandate the use of glucocorticoids at high dose or for prolonged periods of time.

### Magnitude of Clinical Renal Response

A number of studies have shown that failure to attain a very robust reduction of proteinuria (to levels below 0.5–0.8 g/24 h) by 12 months of treatment has been linked to a significantly increased risk for flares [18, 46]. As a matter of fact, the lower proteinuria gets (for instance, as low as 0.15 g/24 h), the higher the odds for sustained remission of LN [47, 48], probably reflecting also a deeper state of immunological and histological remission. In agreement with the aforementioned data, patients with only partial improvement in proteinuria (i.e., not reaching the complete response proteinuria thresholds) are less protected against future flares and progression to CKD or ESKD [49]. Although some of these partial responders might have some degree of “fixed” proteinuria due to irreversible damage in the kidneys, these data are supportive of a “treating-to-target” strategy in LN. To this end, the EULAR together with the ERA-EDTA have proposed that treatment should aim at a reduction of proteinuria by ≥ 25% by 3 months, ≥ 50% by 6 months, and to below 0.5–0.7 g/24 h by 12 months, all coupled with stable/improved eGFR (within 10% of baseline value) [5, 50•]. Notably, the earlier these targets are attained, the better the kidney outcomes [18, 51]. Notwithstanding the fact that a “watchful waiting” strategy is prudent for LN patients who steadily improve so as to avoid over-treatment, these findings suggest that a “hit hard and early” strategy can be beneficial for the prevention of flares and long-term kidney function preservation.

### Abnormal Lupus Serology

The observation that serological abnormalities (low C3/C4, high anti-dsDNA titers) can take longer to improve as compared to clinical activity and also that SLE patients with prolonged serological activity but clinically quiescent disease do not accrue more damage over time has led to the concept that treatment should not be guided by serology [52]. Nevertheless, there is evidence to suggest that “full” clinical and serological remission may be more protective against flares than isolated clinical remission [53, 54]. In the case of LN, a systematic literature review showed that the persistence of hypocomplementemia and/or high anti-dsDNA titers signifies a subgroup of SLE patients at increased risk (2.0 to 3.8-fold) for kidney flares [55•]. Whether this tendency reflects the putative pathogenic role of immunocomplexes or is a surrogate of the lupus disease state remains unknown. Pending more definitive evidence, most experts would advise caution in the withdrawal of immunosuppression in LN patients with persistent, non-improving serological markers.

### The Choice of Immunosuppressive/Biological Treatment

High-quality data from randomized controlled trials (RCTs) in LN have indicated the superiority of certain therapeutic agents in maintaining a durable response. In the ALMS study, responders to induction treatment (with either high-dose intravenous cyclophosphamide or mycophenolate) were randomized to continue with either mycophenolate or azathioprine. Over a 3-year period, patients on the mycophenolate arm experienced significantly fewer relapses as compared to their counterparts on azathioprine [56]. Notably, those who were treated initially with cyclophosphamide and then switched to mycophenolate had a lower rate of flares and treatment failures. Conversely, switches from mycophenolate to azathioprine carry the highest risk for relapses [56], a finding replicated in other trials [20]. More recently, the BLISS-LN trial showed that the addition of belimumab to standard therapy resulted in a significant reduction (by almost 50%) in the rate of renal relapses and other adverse renal events, and this effect was irrespective of the reduction of proteinuria and renal response [15•]. In agreement, a combined analysis of the BLISS datasets (non-renal SLE) revealed that the risk of renal flares was lower among patients receiving intravenous belimumab 10 mg/kg (HR 0.62; 95% CI 0.41–0.92) and intravenous belimumab 1 mg/kg (HR 0.42; 95% CI 0.22–0.79) [57•]. Similar results have not yet been demonstrated for voclosporin or other calcineurin inhibitors, which are known to be associated with rebound increases in proteinuria upon discontinuation. Altogether, and pending confirmation in real-life cohorts, the combination of belimumab with mycophenolate may be the preferred treatment choice for patients at high risk for LN flares or with relapsing LN [7].

### Duration of Immunosuppressive/Biological Treatment

In LN, the optimal duration of treatment remains ill-defined. In the 10-year follow-up of the European Lupus Nephritis Trial, more than half of the patients were still on immunosuppressive treatment. Among patients with proliferative LN, the duration of mycophenolate treatment < 24 months had a hazard ratio of 5.94 for subsequent flare [23]. In an observational study from Italy, patients who withdrew immunosuppressive treatment without flaring tended to have received longer treatment (98.1 versus 31.0 months; *p* = 0.01) and had attained longer remission (52.8 versus 12.0 months; *p* = 0.000) before the withdrawal of therapy as compared to their flaring counterparts. Recently, the WIN-lupus trial evaluated LN patients who had received maintenance with either azathioprine or mycophenolate for 2–3 years and who were randomized (1:1) to immunosuppressive treatment continuation (*n* = 40) or discontinuation (*n* = 44). The study failed to demonstrate non-inferiority since patients in the discontinuation group had more relapses of LN and more extra-renal flares (27.3% versus 12.5%) [58]. To this end, the EULAR/ERA-EDTA recommends for at least 3 to 5 consecutive years of treatment [5]. Depending on the duration of remission, slow gradual tapering of immunosuppressive/biological treatment can be attempted with vigilant follow-up for the early detection of possible flare-ups.

### Use of Hydroxychloroquine

Due to its multifaceted favorable effects, hydroxychloroquine (HCQ) is recommended for all SLE patients [59]. In particular, HCQ use has been linked to reduced rates of exacerbations, and vice versa; flares in SLE patients tend to occur following HCQ discontinuation. The Italian study by Moroni et al. [60] showed that continuing HCQ after withdrawal of immunosuppressive agents was linked to reduced renal flares. Also, in a case series of pediatric LN under a prescribed HCQ dose of 4.0–5.5 mg/kg/day, a HCQ blood cut-off level under 1075 ng/mL was associated with increased flares [61]. The same trends have been described for adults with LN [62]. Finally, in the pooled BLISS dataset analyses by Gomez et al. [57•], the use of antimalarials was protective against renal flares (HR: 0.66; 95% CI: 0.55–0.78). Collectively, these data reiterate the central role of HCQ/antimalarials in the treatment of SLE and LN.

### Adherence to Treatment

The issue of poor compliance to prescribed therapies has been well recognized in patients with SLE and is likely to multiple factors [63, 64]. Not unexpectedly, adherence is associated with increased success of treatment and fewer relapses. Indeed, in a survey of 104 LN patients, non-adherence to medications carried a 3.7-fold increased risk for a single episode of flare and a 4.9-fold increased risk for multiple flare attacks [65]. In this respect, physicians should assess treatment adherence on a regular basis and try to identify possible causes for lower compliance such as those related to patient preferences and beliefs, socio-financial issues, treatment-related harms, polypharmacy, and othesr. Of note, the EULAR/ERA-EDTA recommends that “*in case of failure to achieve the treatment goals, thorough evaluation of the possible causes is recommended, including assessment of adherence to treatment and therapeutic drug monitoring*” [5].

### Residual Activity in Repeat Kidney Biopsy

Monitoring of LN is done primarily on clinical grounds; however, there is often discordance between urinalysis, serological markers, and the underlying kidney histology [66]. In a cohort of 51 patients with complete renal response who underwent a second biopsy, residual low-grade histological activity (NIH activity index [AI] ≥ 2) was revealed in 11 (19.6%) [67]. In these patients, subsequent renal exacerbations were more frequent and occurred at an earlier time point as compared to their counterparts with AI < 2. These results corroborate a previous study where LN patients who had received immunosuppressive treatment for at least 36 months and had been in complete response for at least 12 months underwent a repeat biopsy, and immunosuppression was withdrawn over a period of 6 months [68•]. The presence of histological activity (AI > 2)—especially endocapillary proliferation—could predict the risk of renal flare independent of other clinical predictors. Together, and pending validation in future studies such as the ReBioLup (https://clinicaltrials.gov/ct2/show/NCT04449991), these data suggest that repeat kidney biopsy could be useful to determine the individual risk for flare and/or progression to CKD/ESRD, therefore informing the decision for treatment modification.

## Conclusion

A paradigm shift in the care of SLE is that, in addition to acute control of inflammation, disease stabilization and prevention of flares are critical to reduce patient exposure to glucocorticoids and preserve organ function. This concept is even more relevant in the case of LN, where a vital organ is affected with obvious consequences if not adequately managed, and patients are typically treated with high dose of glucocorticoids. The underlying pathophysiology and interplay between various factors in precipitating LN flares remain ill-defined. The association of certain predictors (demographic, clinical, immunological, and histological) with the occurrence of renal relapses may be reflective of the high inflammatory/autoimmunity burden in these patients and can be considered for initial risk stratification. Notwithstanding the therapeutic implications of such stratification that have not been formally assessed, the expanding treatment armamentarium in LN includes immunosuppressive and biological agents with proven capacity to amplify renal response and prevent flares. Treatment of LN should be viewed as a long-lasting battle, and patients should be informed upfront about the benefit of maintaining treatment for several years, while of course, accounting for their preferences and needs. We anticipate that intensive research in the field of biomarkers, including plasma [69] and urine [70–77] mediators such as TWEAK, VCAM-1, CD163, and matrix metalloproteinases, will be fruitful and provide novel non-invasive tools to monitor renal disease activity, thus enabling prognostication and treatment tailoring in patients with LN.
